# High-Temperature Behavior of Pd/MgO Catalysts Prepared via Various Sol–Gel Approaches

**DOI:** 10.3390/gels10110698

**Published:** 2024-10-27

**Authors:** Grigory B. Veselov, Danil M. Shivtsov, Ekaterina V. Ilyina, Vladimir O. Stoyanovskii, Andrey V. Bukhtiyarov, Aleksey A. Vedyagin

**Affiliations:** Boreskov Institute of Catalysis, 5 Lavrentyev Ave., Novosibirsk 630090, Russia; g.veselov@catalysis.ru (G.B.V.); danil@catalysis.ru (D.M.S.);

**Keywords:** magnesium oxide, sol–gel synthesis, palladium catalysts, CO oxidation, prompt thermal aging, metal–support interactions

## Abstract

A series of Pd/MgO catalysts based on nanocrystalline MgO were prepared via different sol–gel approaches. In the first two cases, palladium was introduced during the gel preparation, followed by drying it in supercritical or ambient conditions. In the third case, aerogel-prepared MgO was impregnated with an ethanol solution of Pd(NO_3_)_2_. The prepared catalysts differ in particle size and oxidation state of palladium. The catalytic performance and thermal stability of the samples were examined in a model reaction of CO oxidation at prompt thermal aging conditions. The as-prepared and aged materials were characterized by low-temperature nitrogen adsorption, UV-vis spectroscopy, X-ray photoelectron spectroscopy, transmission electron microscopy, and ethane hydrogenolysis testing reaction. The highest initial activity (T_50_ = 103 °C) was demonstrated by the impregnated sample, containing Pd^0^ particles of 3 nm in size. The lowest T_50_ value (215 °C) after aging at 1000 °C was demonstrated by the impregnated Pd/MgO-WI sample. The high-temperature behavior of the catalysts was found to be affected by the initial oxidation state and dispersion of Pd. Two deactivation mechanisms, such as the agglomeration of Pd particles and migration of small Pd species into the bulk of the MgO support with the formation of Pd-MgO solid solutions, were discussed.

## 1. Introduction

Magnesium oxide is often considered in the literature as a model support with pronounced basic properties. It has a relatively simple structure, which can be easily simulated. Therefore, it is not surprising that MgO was the subject of several computational studies focused on the type of interactions between the MgO matrix and supported metals [1,2,3]. In addition, in the last decade, MgO was successfully used as a support of the catalysts for various catalytic processes (AuAg-MgO for oxidation of n-octanol [4]; Ru-MgO for ammonia synthesis and decomposition [5,6]; Pd/MgO for CO oxidative coupling to dimethyl oxalate [7] and selective hydrogenation of acetylene [8,9]; VO_x_/MgO for oxidative dehydrogenation of propane [10]; Ni/MgO for CO_2_ methanation [11,12], etc.). Due to its basic properties, magnesium oxide can serve as an efficient carbon dioxide capture system, capable of being regenerated multiple times [13,14,15,16]. MgO-based systems are also commonly applied as catalysts for processes such as steam reforming and carbon dioxide reforming of methane. Among these systems, Ni/MgO is of special interest [3,17,18,19,20,21].

MgO can also be used as a dopant to tune the electronic, acidic, or structural properties of the catalysts. In general, doping with MgO increases the number of basic sites on the surface of the support. However, it can also greatly alter the metal–support interactions, as numerously reported in the literature [22,23,24,25,26]. For instance, MgO affected the basicity and metal–support interactions of Pd/MgO-Al_2_O_3_ catalysts, thus improving the dimethyl oxalate yield during the CO oxidative coupling process [27]. The same explanation was reported by Barzegari et al. [28] when discussing the excellent performance of MgO-doped Ni-SiO_2_ catalysts for propane steam reforming.

It is important to note that MgO influences the performance of Pd-based catalysts in the oxidation processes. Thus, Sinha Majumdar et al. [29] studied the impact of MgO on the CH_4_ oxidation activity of Pd/Al_2_O_3_ and Pd/SSZ-13 catalysts. In both cases, MgO improved the catalytic performance of the samples and their stability at hydrothermal aging. The observed effects were attributed to the influence of Mg during the surface roughening and restructuring on metal–support interactions, which led to higher reducibility of PdO_x_ sites and preferential stabilization of active Pd (100) facets. The higher sintering resistance of MgO-doped Pd-based catalysts was also reported by Zhao et al. [30]. The authors found that MgO improved the catalyst basicity, increased the electron density of metallic Pd, and stabilized PdO against forming inactive Pd(OH)_x_ species.

Due to the relatively extensive use of MgO as a support and dopant, several studies mainly focus on the interactions between MgO and supported metals. As reported in these studies, there are a few types of interactions that can occur between MgO and precious metals like Pd and Pt. Density functional theory (DFT) calculations demonstrate that the shape of metal particles and, as a consequence, the type of active sites on the surface are affected by their interactions with the faces of MgO [31]. Electron transfer may occur between Pd and MgO, thus leading to the formation of charge-separated Pd^δ+^-MgO sites [32]. In some cases, the interaction of supported metal with MgO can lead to the redispersion of large Pd particles into small ones or even single atoms. Tanabe et al. [33] observed that Pt particles of 20 nm in size transformed into smaller Pt particles of 2–5 nm during a sequential oxidation-reduction treatment at 1073 K. They supposed that the formation of Mg_2_PtO_4_-like phase is a driving force for this process. Such a redispersion effect can be used purposefully to prepare single-atom catalysts with a metal concentration of 0.7 at/nm^2^ [34].

Recently, Okumura et al. [35,36] discovered using X-ray absorption spectroscopy that MgO can form Pt-MgO and Pd-MgO solid solutions. In both cases, the precious metals are in the (+4) oxidation state. The temperature required for the complete formation of the Pd-MgO solid solution was found to be in the range of 973–1273 K. Interestingly, when the temperature was increased to 1373 K, a segregation of the solution occurred. The authors assessed the changes in the dispersion of Pd with increasing the calcination temperature via CO chemisorption and revealed that the dependence was non-monotonic. In the case of Pd nanoparticles supported on commercial MgO, the dispersion decreased from 27 to 2% in the range of 573–973 K, then increased to 28% in the range of 973–1173 K, and finally decreased again to 14% at 1373 K. The dependence was even more complex for palladium supported on the MgO treated with boiling water. However, the possible effects of these changes on the catalytic performance of Pd/MgO catalysts remain uncovered.

The present work aimed to reveal the high-temperature behavior of the sol–gel-prepared Pd/MgO catalysts. As discussed previously, materials containing Pd and MgO can find many catalytic applications in the chemical industry. In particular, the data on the high-temperature stability of such systems are important for the preparation of automotive catalysts, which are intended to decrease the environmental pollution level. For this purpose, catalytic experiments were performed in a prompt thermal aging (PTA) mode, consisting of eleven temperature-programmed heating–cooling cycles with a step-by-step increase in the final heating temperature. The reaction mixture used in the catalytic experiments contained CO, hydrocarbons, NO, oxygen, and nitrogen as a balance. The preparation method is based on a sol–gel technique with some variations [8]. This allowed us to obtain Pd nanoparticles of different sizes and oxidation states. A set of methods used to assess the state of supported palladium before and after thermal aging included UV-vis spectroscopy, X-ray photoelectron spectroscopy, transmission electron microscopy, and ethane hydrogenolysis testing reaction. The effects of the oxidation state and the dispersion of Pd on the catalytic performance and thermal stability were elucidated. Finally, the deactivation mechanisms were supposed.

## 2. Results and Discussion

**Figure 1:**
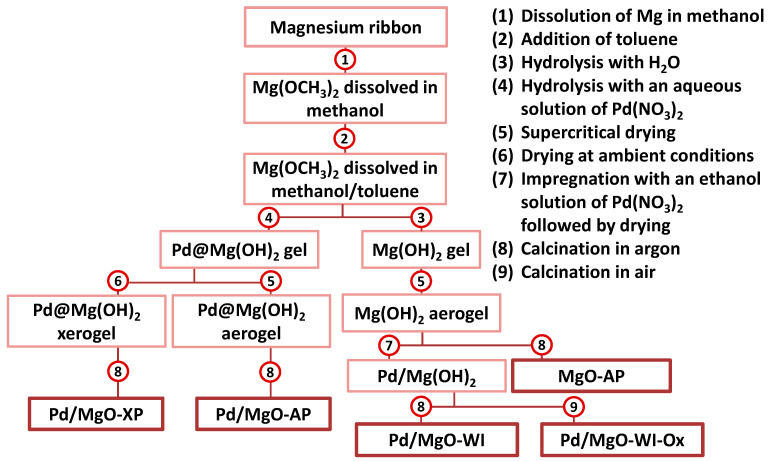
Flowchart of the sol–gel-based methods used to prepare a series of Pd/MgO catalysts.

A series of Pd/MgO catalysts was synthesized via four preparation routes, as depicted in Figure 1. The samples were examined in a model reaction of CO oxidation under prompt thermal aging (PTA) conditions. The results are presented in Section 4.1. The as-prepared and aged samples were characterized by low-temperature nitrogen adsorption/desorption, transmission electron microscopy (TEM), UV-vis diffuse reflectance spectroscopy, X-ray photoelectron spectroscopy, and the testing reaction of ethane hydrogenolysis. These data are collected and discussed in Section 4.2.

### 2.1. High-Temperature Behavior of Pd/MgO Catalysts Under PTA Conditions

**Figure 2:**
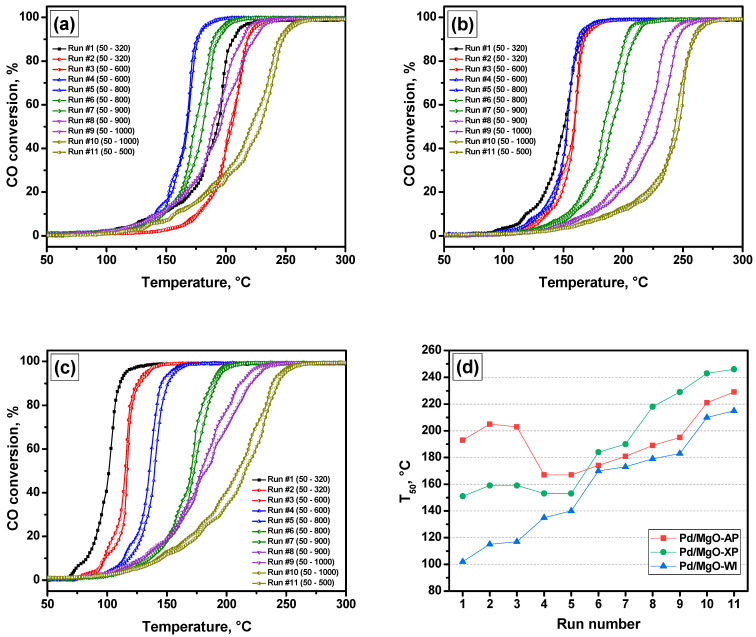
Light-off curves of CO conversion under PTA conditions for the catalysts under study: (**a**) Pd/MgO-AP; (**b**) Pd/MgO-XP; (**c**) Pd/MgO-WI. Dependences of the temperature of 50% conversion of CO (T_50_) on the run number for the same catalysts (**d**).

In order to characterize both the catalytic activity and stability of the Pd/MgO catalysts, they were tested under PTA conditions. Such a technique allows one to follow the changes in the catalytic performance of the samples during consecutive increases in the final temperature of the catalytic heating–cooling cycles performed using a reaction mixture containing CO, hydrocarbons, NO, oxygen, and balanced nitrogen. Figure 2a–c shows the corresponding light-off curves (temperature dependences) of CO conversion for Pd/MgO-AP, Pd/MgO-XP, and Pd/MgO-WI samples. In Figure 2d, the dependences of the temperature of 50% conversion of CO (T_50_) on the number of catalytic cycles (run number) are plotted.

It should be noted that run-to-run deactivation is quite common for Pd-containing catalysts. As considered by many researchers, the main reason for the deactivation of such catalysts is an enlargement of palladium particles at high temperatures [37,38,39,40]. It was recently reported by Okumura et al. [36] that the interaction of palladium and magnesium oxide in an oxidizing atmosphere in the temperature range of 700–1000 °C can proceed with the formation of Pd-MgO solid solutions. At temperatures above 1100 °C, these solutions decompose. In addition, as reported by Chen et al. [41], Pd can migrate into the subsurface layer of MgO upon heating in an oxidative medium. It is obvious that the formation of solid solutions leads to the removal of active metal species from the surface of the catalyst, thus causing a decrease in the catalytic activity. From this point of view, it is of special interest to study the high-temperature behavior of the Pd/MgO catalysts prepared via various sol–gel routes.

As follows from Figure 2, the three samples under consideration differ noticeably in their initial activity. Thus, the Pd/MgO-WI sample exhibits the best initial performance (Figure 2c). However, in the course of the PTA test, a pronounced shift of the light-off curves to the high-temperature region is well seen. It is important to note that a slight decrease in the catalytic activity after the first run is observed for all the samples, which is due to the rearrangement of catalytically active sites under the action of the reaction medium. The Pd/MgO-XP sample, despite a slight decrease in activity after the first run and an increase after the third run, demonstrates relatively stable behavior in the first five runs (Figure 2b). In the case of the Pd/MgO-AP sample (Figure 2a), a significant reactivation effect is observed in the fourth run, which corresponds to the first aging at 600 °C.

The observed differences in the behavior of the samples in the first PTA cycles are related to the different oxidation states of palladium located on the surface of the catalyst. For instance, the highest catalytic activity of the Pd/MgO-WI sample can be assumed to be due to the metallic state of Pd. In such a case, the loss of activity during the PTA test can be related to the oxidation of the dispersed Pd^0^ species, as well as their agglomeration. Within the assumed paradigm, the initial catalytic activity of the Pd/MgO-XP sample is lower due to a higher contribution of oxidized Pd species and, possibly, stronger metal–support interactions. The latter is typical for the highly dispersed Pd^2+^/Pd^0^ species. In the case of the Pd/MgO-AP sample, the largest particle size and the absence of Pd in the metallic state can be the reason for the lowest initial activity.

At temperatures of 800 °C and higher (6–11th runs), the Pd/MgO-AP, Pd/MgO-XP, and Pd/MgO-WI samples show the same run-to-run deactivation behavior but with different deactivation rates. According to the deactivation rate, the samples can be ranked as follows: Pd/MgO-XP >> Pd/MgO-AP > Pd/MgO-WI. As mentioned above, the deactivation of Pd/MgO catalysts can be caused by the agglomeration of metal particles and the formation of Pd-MgO solid solutions. Since the smallest particle size and the strongest metal–support interactions are assumed for the Pd/MgO-XP sample, it can be expected that the formation of Pd-MgO solid solutions is preferable in this case [36]. In addition, if the main mechanism of agglomeration of palladium particles is Ostwald ripening, the smaller particle size should lead to faster particle growth [40]. The Pd/MgO-AP sample assumedly has the largest particle size in the initial state. These large Pd particles can undergo redispersion at 600 °C, thus explaining the observed reactivation effect. This redispersion process probably leads to the appearance of the Pd/PdO species with a decreased crystallite size. In addition, it can be assumed that the deactivation rate can also be affected by the initial oxidation state of palladium.

**Figure 3:**
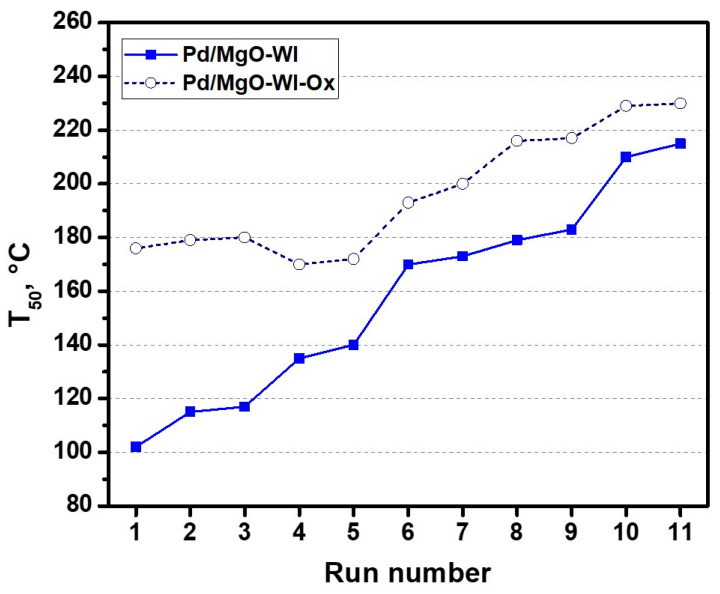
Dependences of the temperature of 50% conversion of CO (T_50_) on the run number for the Pd/MgO-WI and Pd/MgO-WI-Ox samples.

To clarify the above-said assumption, Mg(OH)_2_ aerogel impregnated with palladium precursor was calcined in air at 500 °C, as shown in Figure 1. Note that this sample was denoted as Pd/MgO-WI-Ox. The catalytic performances of the Pd/MgO-WI and Pd/MgO-WI-Ox samples are compared in Figure 3. As shown below, these two samples should contain palladium particles of the same particle size. Therefore, the differences in their catalytic properties could be connected only with the oxidation state of palladium. As seen, the behavior of the Pd/MgO-WI-Ox sample resembles that of the Pd/MgO-AP and Pd/MgO-XP samples. The position of the T_50_ dependence graph for the Pd/MgO-WI-Ox sample is intermediate regarding the same graphs for the two mentioned samples. The Pd/MgO-WI-Ox catalyst also exhibits redispersion at 600 °C. Then, this sample deactivates at a higher rate. Thus, the ΔT_50_ value between Pd/MgO-WI and Pd/MgO-WI-Ox in run 9 is 35 °C, while in run 11, this value is about 13 °C. It is important to mention again that the Pd/MgO-WI and Pd/MgO-WI-Ox samples differ mainly in the oxidation state of palladium. The samples, which initially contain palladium in the form of PdO, are characterized by a lower thermal stability.

### 2.2. Characterization of the As-Prepared and Aged Pd/MgO Catalysts

**Figure 4:**
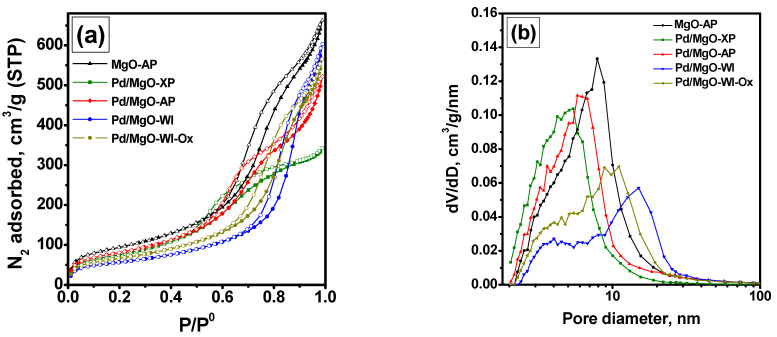
(**a**) Low-temperature (77 K) nitrogen adsorption/desorption isotherms for pure MgO-AP and palladium-containing catalysts. Filled and empty symbols indicate the adsorption and desorption branches, respectively. (**b**) Pore size distributions for pure MgO-AP and palladium-containing catalysts.

The next stage of the research was devoted to clarifying all the assumptions made during the catalytic tests. The porous structure of the support and the prepared Pd-containing catalysts was investigated by low-temperature (77 K) nitrogen adsorption/desorption. The obtained results are given in Figure 4. As seen, the isotherms are characterized by a hysteresis loop of the H3 type, which is characteristic of non-rigid aggregates of plate-like particles (e.g., certain clays) [42]. The same hysteresis loop can also appear if the porous network consists of macropores, which are not filled with the pore condensate completely. The adsorption isotherms for the MgO-AP, Pd/MgO-WI, and Pd/MgO-AP samples show rather narrow hysteresis loops and a rise on the adsorption branch at P/P^0^ close to 1. The latter can be due to the presence of macropores. Contrarily, the Pd/MgO-XP catalyst demonstrates a plateau at high P/P^0^, which is characteristic of Type IVa, according to the International Union of Pure and Applied Chemistry (IUPAC) classification [42]. This suggests that the mesopores predominate in the porous structure of the Pd/MgO-XP sample.

The textural characteristics of the samples, such as specific surface area (SSA), pore volume, and average pore diameter (D_pore_), were calculated using the low-temperature nitrogen adsorption data (Table 1). The maximum SSA value is observed for pure MgO-AP. The introduction of palladium during the sol–gel synthesis decreases this value by 45 and 60 m^2^/g for the Pd/MgO-AP and Pd/MgO-XP samples, respectively. In both cases, a decrease in pore volume is also observed. These effects can be assigned to the influence of metal and nitrate ions on the gel formation processes and the features of the calcination stage [43]. In the case of Pd/MgO-XP, the pore volume decreased more significantly due to the influence of capillary forces that appeared during the drying procedure performed at atmospheric pressure. In the case of aerogel synthesis, the shrinkage of the porous structure is partially avoided. It should be noted that in the Pd/MgO-AP and Pd/MgO-XP samples, pores are mainly represented by mesopores with an average diameter smaller than 10 nm (Figure 2b). For the impregnated Pd/MgO-WI sample, the most significant drop in SSA to 205 m^2^/g, along with a shift of the pore size distribution toward larger values of ~20 nm, is observed. This can be explained by the collapse of a fine porous structure and agglomeration of the support particles.

**Figure 5:**
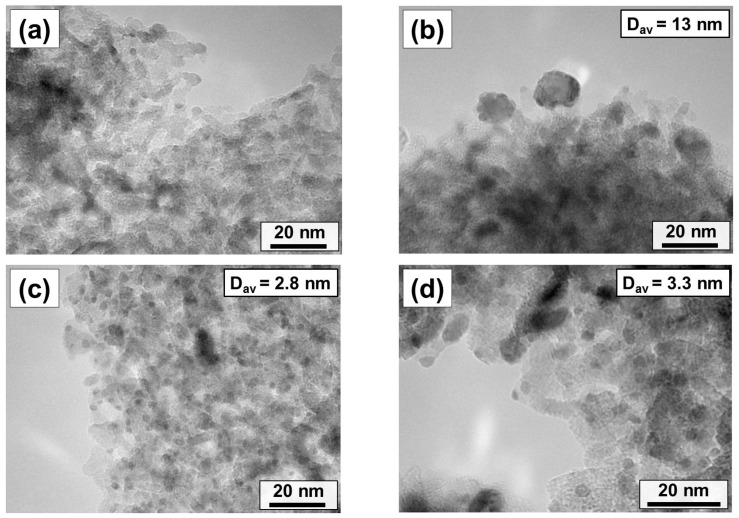
TEM images of the Pd-containing catalysts: (**a**) Pd/MgO-XP; (**b**) Pd/MgO-AP; (**c**) Pd/MgO-WI; (**d**) Pd/MgO-WI-Ox. The average particle size (D_av_) values were calculated from the corresponding particle size distributions. Note that at least 200 particles were measured in each case.

All Pd-containing samples were examined by TEM. The obtained micrographs are presented in Figure 5. In the case of the Pd/MgO-XP sample (Figure 5a), only a small number of palladium particles are found. Therefore, their average sizes could not be estimated. Most probably, palladium in this sample is represented by small clusters or individual ions. Contrarily, in the case of Pd/MgO-AP, palladium particles of 5–25 nm in size are well seen. Based on the particle size distribution (Figure S1a), the average diameter of these particles was defined to be 13 nm. Taking into account the pore radius of the Pd/MgO-AP sample, it can be assumed that a significant part of the palladium particles is located outside the porous structure of the support. This is also evident from the micrograph shown in Figure 5b. Very small particles with sizes less than 5 nm are found in the Pd/MgO-WI sample (Figure 5c and Figure S1b). The average particle size is calculated to be 2.8 nm. As seen from Figure 5d and Figure S1c, the calcination of the Pd/MgO-WI sample in air at 500 °C leads to a slight increase in average particle size to 3.3 nm. Thus, it can be concluded here that the Pd particle sizes estimated from the TEM data are in good agreement with the size effects observed in the catalytic experiments.

UV-Vis spectroscopy is currently a standard technique for assessing the state of palladium species and the size of palladium particles supported on alumina. As reported for Pd/Al_2_O_3_ catalysts, which are characterized by weak metal–support interactions, the structure of small PdO species is already completely formed at the calcination temperatures of ~600 °C [44,45]. Such systems demonstrate a d–d transition band at 450 nm and a metal–ligand charge transfer band at 200 nm. The band gap of bulk PdO is estimated to be 0.8–1.5 eV [46,47]. On the other hand, the asymptotic value of the energy gap width (E_g_) for isolated Pd^2+^ ions in an oxygen environment for Al_2_O_3_ support determined by the Tauc method for direct allowed transitions is ~2.35 eV [48].

In the case of Pd/MgO systems, the situation is more complicated. As shown by Peng et al. [7] and in our recent study [8], the band gap of Pd^2+^ species cannot be used to estimate their size in the general case. This limitation appears due to the following reasons. Unlike Pd/Al_2_O_3_, which is characterized by one structural type of Pd^2+^ species with the charge transfer band at 200 nm, Pd/MgO calcined at ~500 °C can contain at least two types of Pd^2+^ species with the charge transfer bands located at ~243 or ~280 nm. Figure S2 shows the detailed UV-Vis diffuse reflectance spectra of the samples. A set of Gaussian functions was used to approximate these spectra. In the UV-Vis spectra of MgO-based systems, the combination of the above-mentioned charge transfer bands is observed. The contribution of each type depends strongly on the preparation route. Thus, the value of the estimated band gap can be determined by the d–d transition band at 450 nm and by the contribution of the metal–ligand charge transfer bands at 280 nm. This makes it dependent on the structure of Pd^2+^ species. Due to the metal–support interactions, these differences in the coordination environment of Pd^2+^ species tend to decrease as the calcination temperature rises. The main type of Pd^2+^ species for the samples calcined at 800 °C and above is a structural type with a band gap at ~240 nm [8].

**Figure 6:**
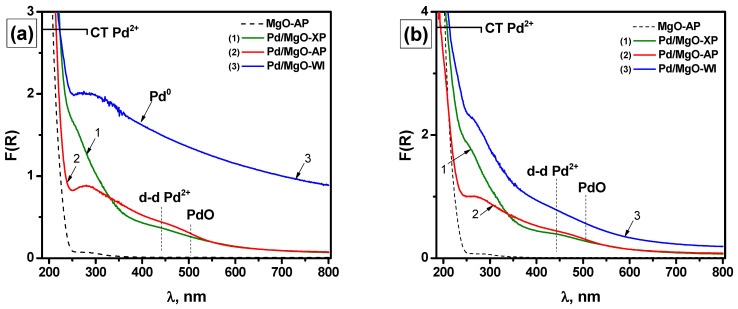
UV-Vis diffuse reflectance spectra of pure MgO-AP and Pd-containing catalysts: (**a**) as-prepared samples; (**b**) after calcination in air at 500 °C for 12 h.

Figure 6a shows the UV-Vis diffuse reflectance spectra of pure MgO and Pd-containing samples (Pd/MgO-XP, Pd/MgO-AP, and Pd/MgO-WI). The spectra of the same samples, which were additionally calcined at 500 °C in the air for 12 h, are given in Figure 6b. The spectrum of the Pd/MgO-WI sample (Figure 6a, spectrum 3) corresponds to metallic palladium (Pd^0^) particles of ~ 3 nm in size at the refractive index of the medium of n ~1.7–1.8, which is close to the known values for MgO. After the calcination at 500 °C in air, the oxidation of Pd/MgO-WI takes place with the formation of Pd^2+^ species in an oxygen environment with the d–d transition band at 450 nm and the metal–ligand charge transfer bands at 200–280 nm (Figure 6b, spectrum 3). However, for the Pd/MgO-XP and Pd/MgO-AP samples, no noticeable changes in the structure of Pd^2+^ species are observed. The charge transfer band at 200 nm is partially overlapped by the fundamental absorption boundary of MgO and, therefore, can only be described qualitatively. In contrast to the Pd/MgO-XP and Pd/MgO-WI samples, the form of Pd^2+^ species with the d–d transition band at 450 nm and the metal–ligand charge transfer band at 280 nm predominates in the case of Pd/MgO-AP (Figure 6 and Figure S2c).

The state of the elements on the surface of the samples was investigated by X-ray photoelectron spectroscopy (XPS). From the analysis of the shape and half-width of the Mg 2p spectrum measured for all samples, it can be concluded that magnesium is represented by two states. The values of the binding energy (BE) of 50.5 and 51.9 eV are characteristic of Mg^2+^ in MgO and Mg(OH)_2_, respectively [49,50]. As an example, Figure S3 shows the Mg 2p spectrum of the Pd/MgO-AP sample. This indicates that magnesium oxide is partially hydrated under the measuring conditions.

**Figure 7:**
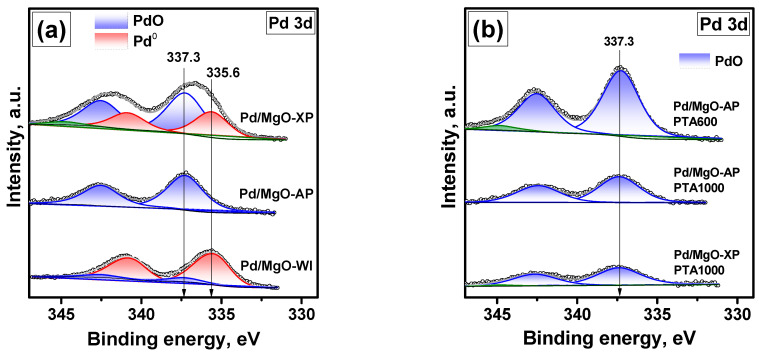
XPS spectra (Pd 3d region) of the Pd-containing samples: (**a**) as-prepared; (**b**) after PTA at various temperatures.

As seen from the analysis of the shape and half-width of the Pd 3d spectra (Figure 7a), palladium can be in one or two states. The corresponding BE values of the Pd3d_5/2_ line are ~335.6 and 337.3 eV. The first one is characteristic of Pd nanoparticles in the metallic state, and the second one corresponds to palladium in the Pd^2+^ state. It should be noted that this BE value is close to that of Pd in palladium hydroxide Pd(OH)_2_ [51].

According to the numerical XPS data presented in Table 2, most of the palladium in the Pd/MgO-WI sample is in the Pd^0^ state. In the Pd/MgO-AP sample, the Pd^2+^ state only is registered. This is consistent with the UV-Vis spectroscopy data. In the case of the Pd/MgO-XP sample, the Pd^0^/Pd^2+^ ratio is about 0.6. At the same time, no metallic state was observed in this sample using the UV-Vis spectroscopy technique. In the Pd/MgO-AP and Pd/MgO-WI samples, the concentration of palladium on the surface is very close to each other, and the Pd/Mg ratio is ~0.004. The surface of Pd/MgO-XP is noticeably enriched with palladium. In this case, the Pd/Mg ratio is as high as 0.007. This confirms the assumption that palladium in Pd/MgO-XP is in the form of dispersed clusters or ionic species.

From the presented data, general conclusions about the state of Pd in the studied samples can be drawn. Palladium in the Pd/MgO-XP sample is represented by a highly dispersed species of < 1 nm in size. In terms of oxidation state, both Pd^0^ and Pd^2+^ are present. In the case of Pd/MgO-AP, larger PdO particles (5–25 nm in size) are located on the outer surface of the MgO support. In the impregnated Pd/MgO-WI sample, metallic particles Pd^0^ of 2–3 nm in size are located inside the pores of the MgO matrix. In the Pd/MgO-Wi-Ox sample, which was calcined in air at 500 °C, the particles are in a state close to PdO, and their size is slightly larger than that for Pd/MgO-WI.

It was of particular interest to examine the samples after the catalytic tests. As mentioned above, the reactivation effect was observed after the aging at 600 °C only for the samples that initially contained palladium in the oxidized state. Figure 7b shows the XPS spectrum of the Pd/MgO-AP sample after PTA at 600 °C. This sample exhibited the most pronounced reactivation effect. As seen from the spectrum, palladium in this sample, after aging at 600 °C, is still in the Pd^2+^ state. At the same time, the intensity of the Pd line is higher. The Pd/Mg ratio increased from 0.0038 for the as-prepared sample to 0.0069 for the PTA600 sample (Table 2). Such an effect of redispersion of initially large palladium particles is well-known for Pd/Al_2_O_3_ and Pd/Ce_0.5_Zr_0.5_O_2_ systems [38,39]. As reported recently, Pd/MgO catalysts can behave very similarly. For instance, Sarma et al. [34] demonstrated that the thermal treatment of PdPt/MgO catalysts in an oxidizing atmosphere at 800 °C leads to the redispersion of metals to their atomic state. Under the conditions used in the present study, the redispersion is observed at 600 °C. It is known that the stabilization of palladium on oxide supports takes place with the participation of surface electron-donor sites [52]. Most probably, the presence of a large number of such sites, along with various defects on the surface of the sol–gel-prepared MgO, facilitates the redispersion of palladium in the oxidizing atmosphere [53].

It is also worth noting that for the Pd/MgO-XP, Pd/MgO-WI-Ox, and Pd/MgO-AP samples, the T_50_ value decreased after the fourth catalytic run by 6, 10, and 36 °C, respectively. As shown above, the initial particle size of palladium increases in the same sequence. Thus, it is obvious that the larger the initial particle size, the greater the effect of redispersion. The aging of the Pd/MgO-XP and Pd/MgO-AP samples at 1000 °C does not change the oxidation state of Pd (Figure 7b) but decreases the Pd/Mg ratio to 0.0031 and 0.0047, respectively (Table 2). This explains the difference in the final activity of these samples. It should be noted that no Pd^4+^ species were registered by XPS. However, Okumura et al. [36] reported that palladium in Pd-MgO solutions is in the (+4) oxidation state. This is supported by the calculations performed by Chen et al. [41]. Okumura et al. [36] also mentioned that the formation of Pd-MgO was observed at the Pd concentration of 0.2 wt% only. It is apparent that, in our case, a large portion of Pd is still on the MgO surface. On the other hand, this does not exclude the possibility that the migration of Pd can contribute to the deactivation process. It is also important to note that there are no reports in the literature on direct observation of Pd^4+^ in Pd/MgO catalysts by XPS.

**Figure 8:**
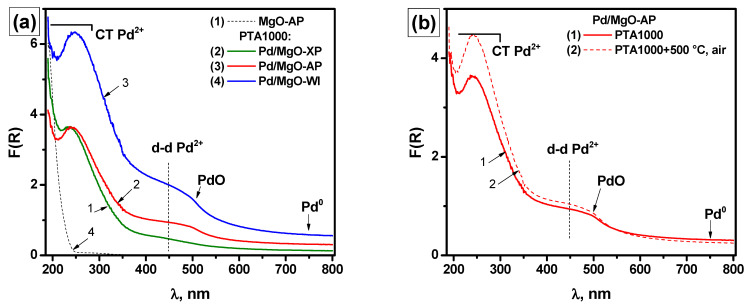
UV-vis diffuse reflectance spectra of Pd-containing samples: (**a**) after PTA at 1000 °C; (**b**) Pd/MgO-AP after PTA at 1000 °C and additional calcination at 500 °C for 6 h.

The UV-Vis spectra of the samples aged at 1000 °C are collected in Figure 8. By comparing the samples on a qualitative level, it is seen that the differences in the charge transfer band region are not as significant as before the aging (Figure 8a). An enlargement of PdO particles is most noticeable for the Pd/MgO-AP and Pd/MgO-WI samples. This is evident from the formation of a characteristic edge of the d–d band in the region of 500 nm. A small fraction of reduced Pd^0^ is also detected. As demonstrated for Pd/MgO-AP (Figure 8b), after additional calcination of the aged sample at 500 °C for 6 h, the intensity in the region of the charge transfer band increases. Along with this, a decrease in the region near 750 nm, which is assigned to Pd^0^ particles, is observed. All these changes in the spectrum testify to the oxidation of highly dispersed Pd^0^ particles.

Figure S4 presents the analysis of the UV–Vis diffuse reflectance spectra of the samples after PTA at 1000 °C with the curve fitting by a Gaussian function. The corresponding plots of (F(R)·E)^2^ depending on the photon energy (E), which characterize the E_g_ values for direct allowed transitions, are also given in Figure S4. The E_g_ values of PdO species determined from these spectra lie in the range of 2.24–2.32 eV.

All the spectra contain the d–d transition band at ∼450 nm and the metal–ligand charge transfer band at 243 nm. Among these samples, Pd/MgO-WI is the only sample whose spectrum demonstrates a mixture of the charge transfer bands located at 243 and 280 nm, similar to the as-prepared samples (Figure S4b).

Based on the E_g_ values, it can be assumed that the dispersion of Pd species increases in the following sequence: Pd/MgO-WI < Pd/MgO-AP < Pd/MgO-XP. However, these differences in dispersion are rather small. It is important to note that the intensity of the UV-vis spectra decreases in the same sequence. This can be due to the different localizations of palladium, which can be localized in bulk or on the surface. By following this interpretation, the palladium concentration on the surface increases in the row: Pd/MgO-XP < Pd/MgO-AP < Pd/MgO-WI. This observation agrees well with the catalytic results, thus confirming the above-said assumptions. Therefore, the differences in the catalytic activity of the aged samples are mainly connected with the different rates of palladium migration into the bulk of MgO.

**Figure 9:**
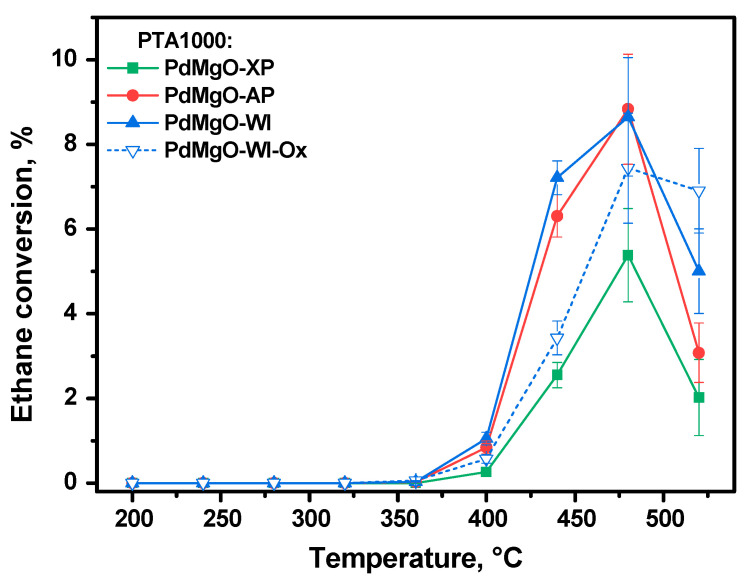
Light-off curves of ethane hydrogenolysis over the Pd-containing catalysts after PTA at 1000 °C.

In order to evaluate the dispersion of palladium in supported catalysts of different compositions, the method based on the test reaction of ethane hydrogenolysis was developed and successfully applied [52,54]. This reaction proceeds on Pd in the metallic state at temperatures of 280 °C and above. As opposed to conventional chemisorption techniques, this method is applicable for the detection of ultra-low concentrations of metal. Light-off curves of ethane conversion in the hydrogenolysis reaction are shown in Figure 9. The analysis of the light-off curve sections in the temperature range of 350–425 °C gives us the possibility to rank the samples in terms of the available surface area of palladium, which correlates with its dispersity. Thus, the dispersity of palladium increases in the following order: Pd/MgO-XP < Pd/MgO-WI-Ox < Pd/MgO-AP < Pd/MgO-WI. These data also support the above-said assumptions and correlate well with the results of other characterization techniques.

**Figure 10:**
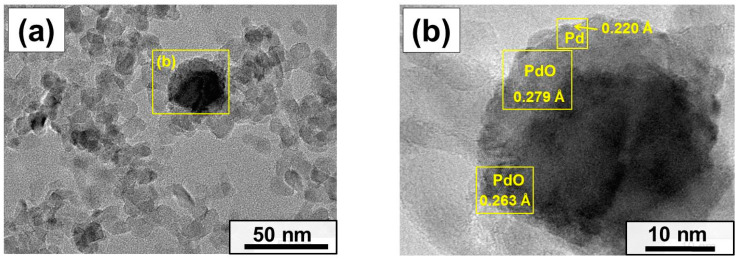

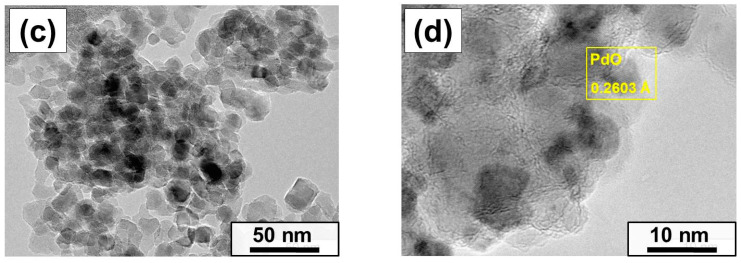
HR TEM images of the samples after PTA at 800 °C: (**a**,**b**) Pd/MgO-AP; (**c**,**d**) Pd/MgO-XP.

It should be emphasized that the aging of the catalysts at 1000 °C reflects their final deactivated state. On the other hand, it was interesting to obtain some information regarding the state of palladium at the beginning of the deactivation process. Therefore, the samples after PTA at 800 °C were explored by high-resolution TEM (HR TEM). The images are shown in Figure 10. In the case of the Pd/MgO-AP sample, PdO particles of ~20–30 nm in size are mainly located on the surface of the MgO support (Figure 10a). Based on the interplanar distances (Figure 10b), Pd nanoparticles of ~2–3 nm in size can be distinguished on the surface of large PdO particles. For the Pd/MgO-XP sample, areas enriched with palladium were found (Figure 10c). These areas are relatively small and do not exceed 10 nm in length. Figure 10d illustrates the magnified image of such an area. The measured interplanar distances correspond to PdO.

It is worth noting that the presented data correspond to the seventh catalytic run, in which the activity of the Pd/MgO-AP sample is higher than that of Pd/MgO-XP. In the further runs, the activity sequence remained the same. Taking into account the HR TEM data, it can be assumed that in the case of Pd/MgO-XP, the initially stronger metal–support interaction decelerates the process of palladium agglomeration. On the other hand, it also causes a descent in catalytic activity. At higher temperatures, the Pd/MgO-XP sample seems to be more prone to the formation of the Pd-MgO solid solution.

**Figure 11:**
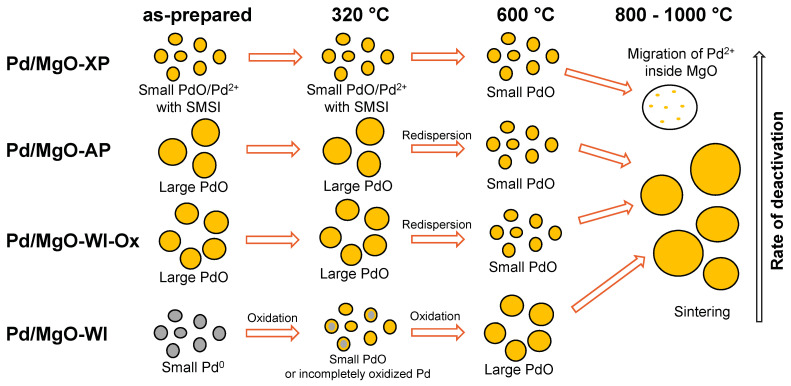
Schematic illustration of the high-temperature behavior of Pd/MgO catalysts.

Summarizing all the obtained results on the high-temperature behavior of the sol–gel-prepared Pd/MgO catalysts, the following findings can be stated. As already mentioned, the deactivation of palladium at high temperatures in the case of Pd-MgO systems can be associated with the agglomeration of palladium particles. Another possible reason is that Pd and MgO can interact strongly with each other. For instance, Pd can migrate into the subsurface layer or bulk of MgO, thus forming Pd-MgO solid solutions. The assumed mechanisms of deactivation are illustrated schematically in Figure 11. In the case of the Pd/MgO-AP sample, as revealed by HR TEM performed after PTA at 800 °C, the formation of large Pd particles (~20–30 nm in size) occurred. For the Pd/MgO-XP sample, some areas enriched with palladium were observed. At the same time, no agglomerated Pd particles were found. In terms of catalytic performance, the Pd/MgO-XP sample aged at 800 °C exhibits worse activity (highest T_50_ values) compared to the Pd/MgO-AP sample aged at the same conditions. Apparently, this is because the strong metal–support interaction (SMSI) decreases the specific activity of palladium. The characterization of the Pd/MgO-XP and Pd/MgO-AP samples after PTA at 1000 °C showed that the surface concentration of palladium in Pd/MgO-XP is lower, which testifies toward the formation of Pd-MgO solid solutions. It should be noted that such solutions cannot be observed directly by physicochemical methods used in the present research, and, therefore, their formation can be judged only implicitly.

According to the UV-Vis spectroscopy and ethane hydrogenolysis data, the Pd/MgO-WI sample showed the highest activity after PTA at 1000 °C and the highest surface concentration of palladium. Presumably, the initial metal particles in this sample are characterized by weak metal–support interactions, and only a minor part of palladium can form solid solutions. The data obtained for the Pd/MgO-WI-Ox sample confirmed the hypotheses about the influence of the initial oxidation state on the deactivation of Pd/MgO catalysts.

## 3. Conclusions

In this research, a series of Pd/MgO catalysts were prepared via various sol–gel routes and characterized by physicochemical methods. The high-temperature performance of the catalysts was examined in a model reaction of CO oxidation in the presence of hydrocarbons under prompt thermal aging conditions. It was found that small nanoparticles (2–3 nm) of metallic Pd supported on the aerogel-prepared MgO support via an incipient wetness impregnation demonstrate the highest activity in CO oxidation. Contrarily, large particles of PdO (10–25 nm) introduced into the MgO matrix via the aerogel technique exhibit the worst catalytic performance. An intermediate activity is demonstrated by highly dispersed Pd^2+^/Pd species distributed within the xerogel MgO matrix.

Since all the studied Pd/MgO catalysts contain palladium in different initial states, their high-temperature behavior in an oxidative medium differs as well. According to the proposed scheme, the oxidation of small Pd particles monotonically decreases their activity in consecutive catalytic runs. The effect of redispersion of large PdO particles supported on MgO was found to take place at 600 °C. The formation of small Pd species noticeably improves the catalytic performance. The larger the initial size of PdO particles, the more significant the effect of redispersion.

Finally, the high-temperature behavior of the Pd/MgO catalysts in the range of 800–1000 °C was found to be influenced by the initial oxidation state of Pd and the dispersion of Pd species. Two deactivation mechanisms, such as agglomeration of Pd particles and migration of Pd into the bulk of MgO followed by the formation of Pd-MgO solid solution, were discussed. Supposedly, higher dispersion and higher oxidation state of palladium favor the latter route. In fact, the Pd/MgO-WI sample prepared via an incipient wetness impregnation and containing small particles of metallic Pd demonstrates the best thermal stability due to the above-said reasons. In the Pd/MgO-XP sample, which contains dispersed Pd^2+^/Pd^0^ species, the deactivation process proceeds at the highest rate. Thus, the main strategy to improve the catalytic activity of Pd/MgO catalysts is the preliminary Pd reduction in hydrogen. Such a treatment increases the initial activity of the catalysts as well as their thermal stability. The particle size of palladium in the as-prepared catalysts should preferably be within the range of 2–3 nm.

## 4. Materials and Methods

### 4.1. Materials

Methanol (J.T. Baker, Phillipsburg, NJ, USA), toluene (Component-Reactive, Moscow, Russia), ethanol (Sigma-Aldrich, Burlington, MA, USA), magnesium ribbon (Aldrich, Burlington, MA, USA), and 14.89 wt% Pd(NO_3_)_2_ solution (Krastsvetmet, Krasnoyarsk, Russia) were used as-received, without further purification. All gases (argon, nitrogen, hydrogen, and helium) were supplied by LLC Chistiye Gazi Plus, Novosibirsk, Russia.

### 4.2. Preparation of the Materials

The Pd/MgO catalysts were prepared via various sol–gel approaches, as described elsewhere [8]. The sample labeled as Pd/MgO-AP was prepared using an aerogel approach. In this case, a metallic magnesium ribbon (1.2 g) was dissolved in methanol (30 mL). Toluene (150 mL) was added to the solution as a stabilizer. The formed magnesium methoxide was hydrolyzed dropwise with water (1.8 mL) at room temperature for 16 h. An ethanol solution of Pd(NO_3_)_2_ was added dropwise to the homogeneous magnesium hydroxide gel. Ethanol solution is required to reduce the amount of water in the magnesium hydroxide gel, which is stabilized by the organic phase. The obtained gel was dried under supercritical conditions. To do this, the gel was placed into an autoclave (AmAr Equipments Ltd., Mumbai, India) and heated up to 265 °C for 3 h. Finally, the autoclave was degassed to remove the solvent. Along with this, the pressure in the autoclave reached 50 atm. The dried aerogel was calcined under an argon atmosphere in a tubular reactor (Zhengzhou Brother Furnace Co., Ltd., Zhengzhou, Henan, China) at 500 °C for 5 h and maintained at this temperature for 3 h. The flow rate of Ar passed through the reactor was 16 L/h.

In the second case, a xerogel approach was applied to prepare the sample labeled as Pd/MgO-XP. The main synthesis steps were the same as in the previous case, excluding the supercritical drying procedure. Here, the obtained gel was dried in air at 100 °C for 16 h. The dried xerogel was calcined in a tubular reactor under the same conditions as Pd/MgO-AP.

The next sample labeled Pd/MgO-WI was synthesized via an incipient wetness impregnation of the aerogel-prepared magnesium hydroxide Mg(OH)_2_-AP. The latter was obtained using the same procedures as in the case of Pd/Mg-AP but without adding palladium nitrate. Mg(OH)_2_-AP was impregnated with an ethanol solution of Pd(NO_3_)_2_. Here, the use of ethanol as a solvent allowed us to minimize the shrinkage of the porous structure due to the less surface tension. The impregnated magnesium hydroxide was then calcined under an argon atmosphere in the same tubular reactor at 500 °C for 5 h and maintained at this temperature for 3 h. The flow rate of Ar passed through the reactor was 16 L/h. In order to prepare a pure MgO-AP sample, Mg(OH)_2_-AP was calcined under the same conditions. The sample labeled Pd/MgO-WI-Ox was prepared via calcination of Mg(OH)_2_ impregnated with Pd(NO_3_)_2_ in air instead of argon.

### 4.3. Physicochemical Characterization Methods

The textural characteristics of the samples were studied by low-temperature nitrogen adsorption/desorption at 77 K. The isotherms were recorded using a Sync 200 automated adsorption analyzer (3P Instruments, Odelzhausen, Germany). Before measurements, the samples were degassed in a vacuum (<1 Pa) at 300 °C for 3 h. The calculations were performed using 3P Sync software (version 10.03.08.00). The specific surface area (SSA) was determined by the Brunauer–Emmett–Teller (BET) method in the P/P_0_ range from 0.07 to 0.23 [55]. The pore volume was calculated at a final relative pressure of 0.990. The adsorption branch of the isotherms was used to calculate the pore size distribution.

The morphology of the as-prepared catalysts was studied by transmission electron microscopy (TEM) using a Hitachi HT7700 TEM microscope with a tungsten source at an accelerating voltage of 100 kV (Hitachi Ltd., Tokyo, Japan). Before examination by TEM, the samples were suspended in ethanol and then deposited on a grid coated with a perforated carbon film. High-resolution TEM (HR TEM) studies of the aged samples were carried out using a JEM-2010CX instrument (Jeol, Tokyo, Japan).

UV-Vis diffuse reflectance spectra were recorded between 190 and 800 nm using a UV-vis spectrometer Cary 300 (Agilent, Santa Clara, CA, USA) and DRA-CA-3300 integrating sphere with the Spectralon standard as a reference. The samples were ground in a mortar to the powdered state before the measurements. The UV-Vis spectra were transformed into the Kubelka–Munk function F(R) [56]. In order to estimate the size of PdO particles, the band-gap width (E_g_) values were determined using the Tauc method. For direct transitions, the graphs of [*F*(*R*_∞_) × *hν*]^2^ versus *hν (Tauc plot)*, where *F*(*R*_∞_) is the Kubelka–Munk function for an infinitely thick sample and *hλ* is the energy of the incident photon, were fitted to a sum of an arctangent curve.

X-ray photoelectron spectra (XPS) were recorded on a SPECS photoelectron spectrometer (SPECS GmbH, Berlin, Germany) using a MgK_α_ radiation (hν = 1253.6 eV; 150 W). The scale of binding energies (BE) was preliminarily calibrated by the position of the peaks of gold and copper: Au4f_7/2_ at 84.0 eV and Cu2p_3/2_ at 932.67 eV. Residual gas pressure during the measurements did not exceed 8 × 10^−9^ mbar. The samples were studied without prior treatment. In the survey spectra of all samples, only lines related to magnesium, palladium, carbon, and oxygen were observed. Other lines were not detected within the sensitivity of the XPS method. The Mg 2p, C 1s, Pd 3d, and O 1s regions were recorded on the surface of the samples to determine the chemical (charge) state and the ratio of atomic concentrations of the elements. To calibrate the measured spectra, the Mg 2p line (BE = 50.5 eV) of magnesium in MgO was used as an internal standard [49,50]. Integral line intensities were measured from the areas of the respective regions (Mg 2p, C 1s, Pd 3d, and O 1s). The relative content of elements on the surface of the samples and the ratio of their atomic concentrations were determined by the integral intensities of photoelectron lines corrected for the corresponding atomic sensitivity coefficients [57]. Processing of the obtained spectral information was carried out using the XPSPeak 4.1 software.

The available surface area of Pd on the surface of the catalysts was estimated by the test reaction of ethane hydrogenolysis as described elsewhere [52,54]. The samples were studied after PTA without any additional pretreatment. The specimen of the catalyst (100 mg; a fraction of 0.25–0.5 mm) was fixed inside the quartz flow-through reactor. At the beginning of the testing experiment, the helium flow was mixed with hydrogen, and the H_2_/He mixture was passed through the reactor for some time until the system reached a steady state. At this point, ethane was added to the flow and passed for 3 min. Then, a sample of the outlet gas mixture was taken for chromatographic analysis (chromatograph Crystal 2000M, Chromatec Instr., Yoshkar-Ola, Russia), and the ethane flow was stopped. For 10 min of the chromatographic analysis, the sample was purged by the H_2_/He mixture in order to regenerate the initial steady state of the sample. This procedure was repeated 5 times at each temperature point within the studied temperature range of 200–520 °C with a step of 40 °C.

### 4.4. Testing the Catalytic Performance at Prompt Thermal Aging Conditions

The high-temperature behavior of the catalysts was studied under prompt thermal aging (PTA) conditions. The experimental setup consists of a gas supply system, a quartz reactor, analysis and registration modules, and instruments that provide regulation and control of the main process parameters, such as temperature and flow rate of gases. For the testing, the sample (fraction of 0.25–0.5 mm) was loaded into the reactor. Then, a reaction mixture containing 1500 ppm of CO, 30 ppm of CH_4_, 40 ppm of C_3_H_6_, 11 ppm of toluene, 14% of O_2_, and balance N_2_, was passed through the reactor. The total flow rate was 334 mL/min.

The PTA test consists of eleven temperature-programmed heating–cooling runs. The initial temperature in all cases was 50 °C, while the final temperature of the runs was varied as follows: 320 °C for the first and second runs; 600 °C for the third and fourth runs; 800 °C for the fifth and sixth runs; 900 °C for the seventh and eighth runs; 1000 °C for the ninth and tenth runs; and 500 °C for the eleventh run. The temperature ramping rate was 10 °C/min. CO concentration in the reaction mixture at the reactor outlet was measured using a ULTRAMAT 6 gas analyzer (Siemens, Munich, Germany) equipped with an infrared cell. CO conversion (X_CO_) was calculated according to Equation (1).

(1) $$X_{CO}\left(\%\right)=\frac{100\cdot (C_{0}-C)}{C_{0}}$$

where *C*_0_ and *C* are the initial and current CO concentrations, respectively. To follow the changes in the catalytic performance of the samples during the thermal treatment, the temperature of 50% CO conversion (T_50_) was used. The error in determining this parameter did not exceed 2 °C.

The samples taken from the reactor after several PTA runs were labeled as follows: PTA600—after the fifth run; PTA800—after the seventh run; and PTA1000—after the eleventh run.

## Figures and Tables

**Table 1:** Textural characteristics of the samples.

Sample	SSA, m^2^/g	Pore Volume, cm^3^/g	D_pore_ *, nm
MgO-AP	340	1.03	8
Pd/MgO-AP	295	0.81	7
Pd/MgO-XP	280	0.53	6
Pd/MgO-WI	205	0.93	15
Pd/MgO-WI-Ox	240	0.88	11

*—average pore diameter.

**Table 2:** Ratios of atomic concentrations of the elements on the surface of the Pd-containing catalysts.

Sample	Pd/Mg	O/Mg	C/Mg	Pd^0^/Pd^2+^
Pd/MgO-XP	0.0070	1.3	0.42	0.6
Pd/MgO-AP	0.0038	1.4	0.54	- *
Pd/MgO-WI	0.0040	1.4	0.19	5.5
Pd/MgO-AP PTA600	0.0069	1.5	0.73	- *
Pd/MgO-AP PTA1000	0.0047	1.6	1.04	- *
Pd/MgO-XP PTA1000	0.0031	1.6	0.87	- *

*—no metallic palladium is detected.

## Data Availability

Data are contained within the article.

## Supplementary Materials

The following supporting information can be downloaded at https://www.mdpi.com/article/10.3390/gels10110698/s1, Figure S1: Pd particle size distributions: (a) Pd/MgO-AP: (b) Pd/MgO-WI; (c) Pd/MgO-WI-Ox; Figure S2: UV-vis diffuse reflectance spectra with the curve fitting by a Gaussian function for the Pd-containing samples calcined in air at 500 °C: (a) Pd/MgO-XP; (b) Pd/MgO-WI; (c) Pd/MgO-AP; Figure S3: XPS spectrum of the Pd/MgO-AP sample in the Mg 2p region; Figure S4: UV-vis diffuse reflectance spectra with the curve fitting by a Gaussian function for the Pd-containing samples after PTA at 1000 °C and additional calcination in air at 500 °C: (a) Pd/MgO-XP; (b) Pd/MgO-WI; (c) Pd/MgO-AP. Corresponding dependencies of (F(R)·E)^2^ on the photon energy (E) characterizing the band-gap width (E_g_) values for direct allowed transitions are also presented.
