# Supplementary material for: High-Temperature Behavior of Pd/MgO Catalysts Prepared via Various Sol–Gel Approaches
Source: Gels. 2024 Oct 27;10(11):698. doi: 10.3390/gels10110698 (PMC11593542; doi:10.3390/gels10110698)
Supplement: Supplementary file 1 [file gels-10-00698-s001.zip › gels-3281729-supplementary.pdf]

gels

MDPI

Supplementary Materials

High-Temperature Behavior of Pd/MgO Catalysts Prepared via Various Sol–Gel Approaches

Grigory B. Veselov, Danil M. Shivtsov, Ekaterina V. Ilyina, Vladimir O. Stoyanovskii, Andrey V. Bukhtiyarov and Aleksey A. Vedyagin *

Boreskov Institute of Catalysis, 5 Lavrentyev Ave., Novosibirsk 630090, Russia
* Correspondence: vedyagin@catalysis.ru

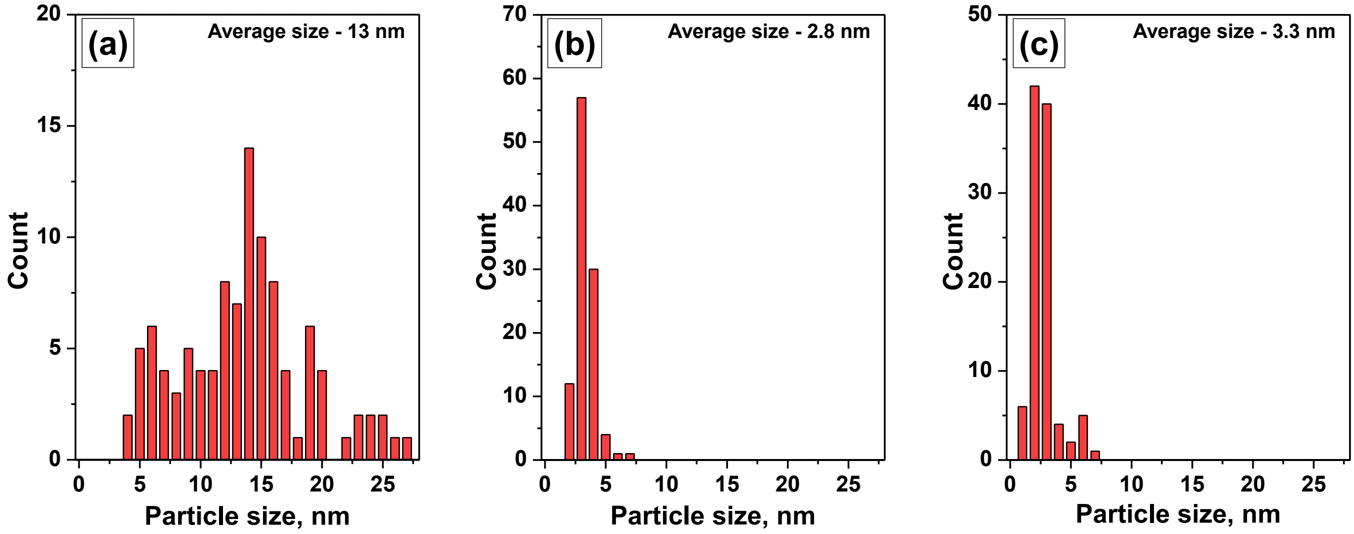

Figure S1. Pd particle size distributions: (**a**) Pd/MgO-AP: (**b**) Pd/MgO-WI; (**c**) Pd/MgO-WI-Ox.

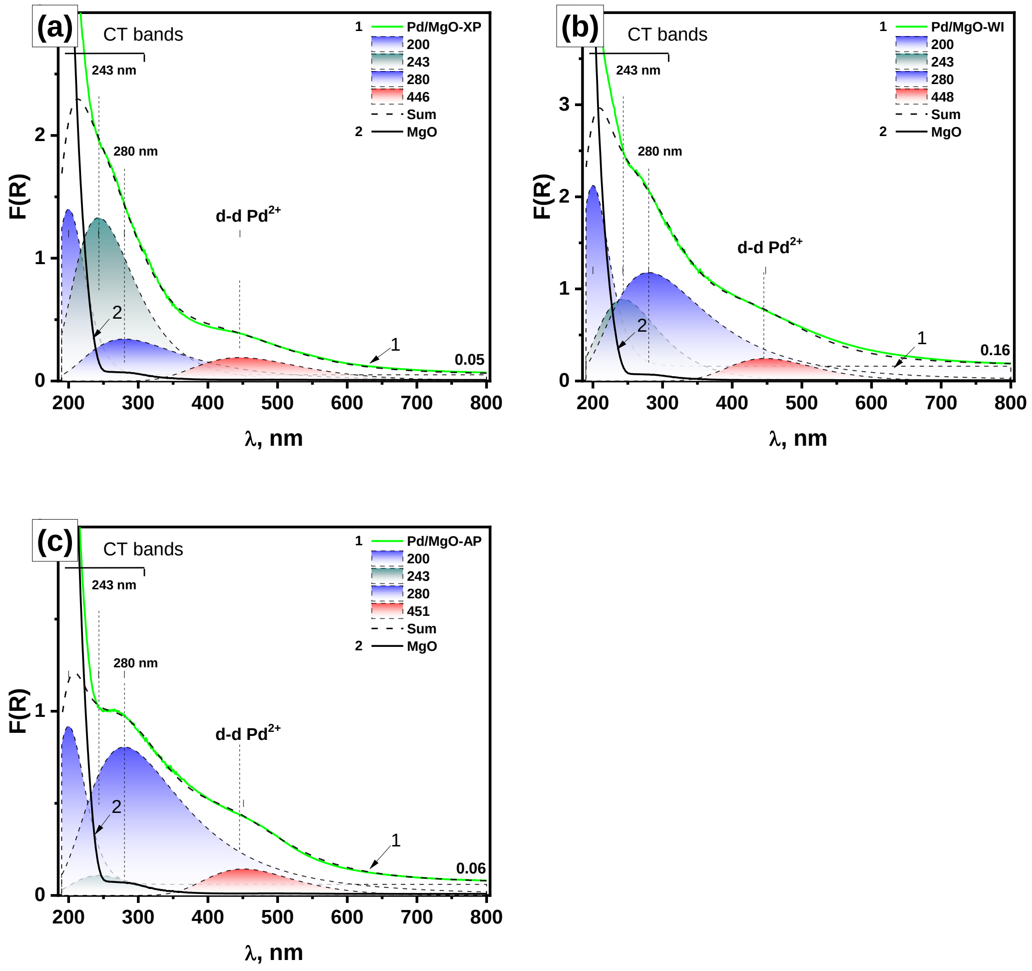

Figure S2. UV-vis diffuse reflectance spectra with the curve fitting by a Gaussian function for the Pd-containing samples calcined in air at 500 °C: (**a**) Pd/MgO-XP; (**b**) Pd/MgO-WI; (**c**) Pd/MgO-AP.

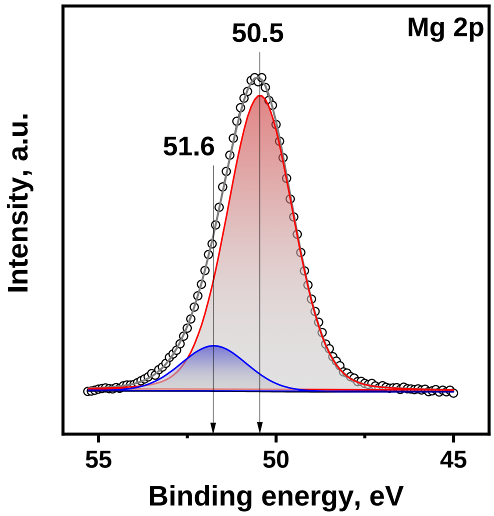

Figure S3. XPS spectrum of the Pd/MgO-AP sample in the Mg 2p region.

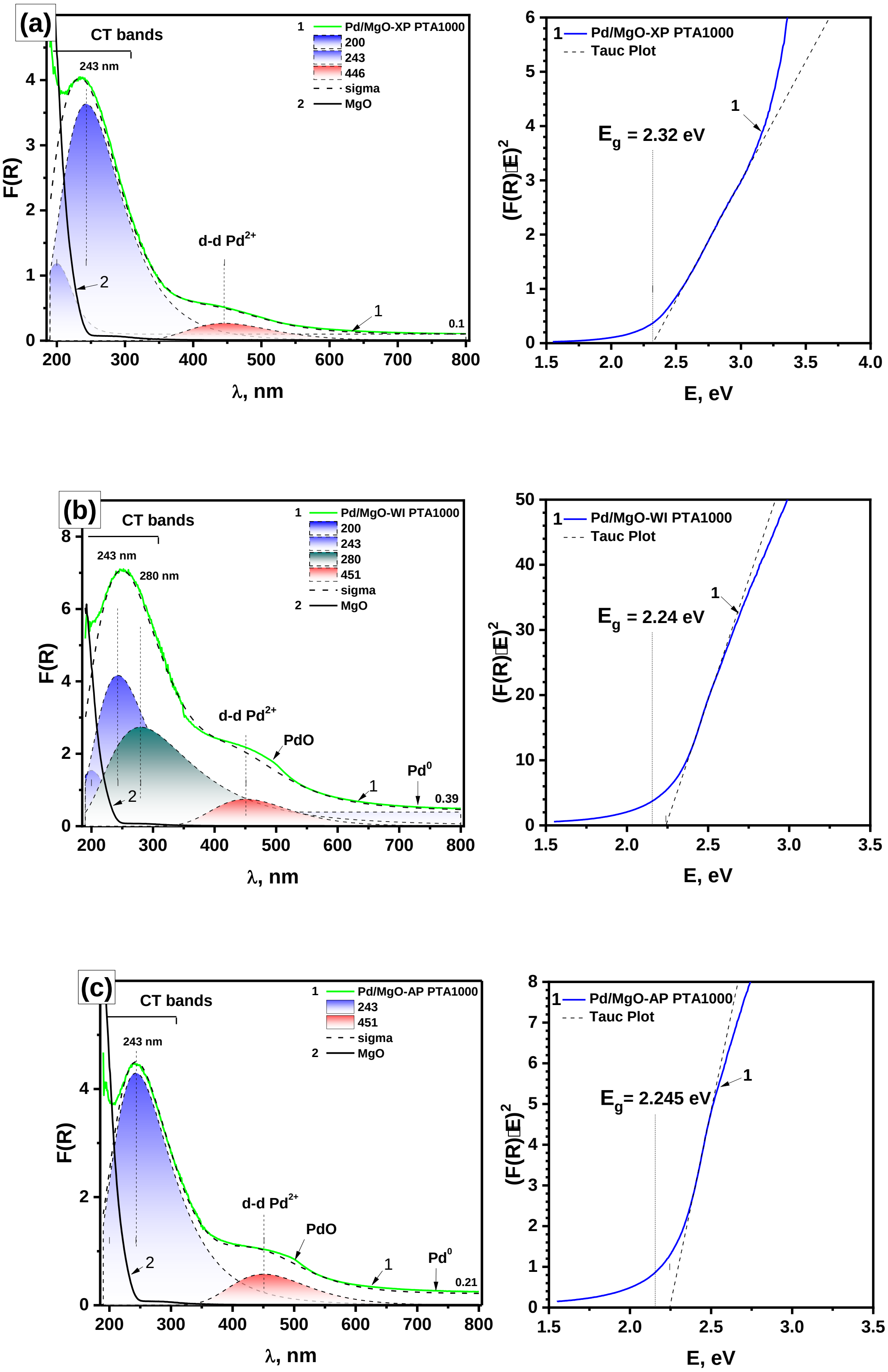

Figure S4. UV-vis diffuse reflectance spectra with the curve fitting by a Gaussian function for the Pd-containing samples after PTA at 1000 °C and additional calcination in air at 500 °C: (**a**) Pd/MgO-XP; (**b**) Pd/MgO-WI; (**c**) Pd/MgO-AP. Corresponding dependencies of $(F(R)\cdot E)^2$ on the photon energy (E) characterizing the band-gap width (E_g) values for direct allowed transitions are also presented.
